# Hierarchical machine learning model integrating clinical history and nursing observations for predicting violent behavior in hospitalized schizophrenia patients

**DOI:** 10.3389/fpsyt.2025.1644341

**Published:** 2025-09-22

**Authors:** Xianfeng Meng, Liang Wang, Ying Duan, Gang Zhu, Jinhuan Wang, Ying Sun, Mingtao Wang, Miao Liu, Chenhui Sun, Longlong Pang, Kunyuan Hu, Wei Yang, Wei Shao, Jintao Ren, Xiaojun Shao, Yang Zhang

**Affiliations:** ^1^ Liaoning Provincial Mental Health Center, Tieling, Liaoning, China; ^2^ Liaoning Maternal and Child Health Hospital, Shenyang, Liaoning, China; ^3^ Department of Psychiatry, The First Affiliated Hospital of China Medical University, Shenyang, Liaoning, China; ^4^ Key Laboratory of Networked Control Systems, Chinese Academy of Sciences, Shenyang, China; ^5^ Shenyang Institute of Automation, Chinese Academy of Sciences, Shenyang, China; ^6^ University of Chinese Academy of Sciences, Beijing, China; ^7^ Shengjing Hospital of China Medical University, Shenyang, Liaoning, China

**Keywords:** schizophrenia, violent behavior, predictive models, machine learning, hierarchical model, risk factors

## Abstract

**Objective:**

To develop and validate a hierarchical machine learning model integrating static clinical features and dynamic behavioral assessments for accurately predicting violent behaviors among hospitalized schizophrenia patients.

**Methods:**

This retrospective study included 346 schizophrenia patients hospitalized from July 2021 to July 2024 in Liaoning Province. Patients were categorized into violent (*n* = 123) and non-violent (*n* = 223) groups based on documented aggressive incidents. Eighteen static clinical variables (e.g., age, gender, history of violence, manic symptoms) were extracted from electronic medical records, and 39 dynamic behavioral indicators (e.g., anger expression, insomnia, auditory hallucinations) were assessed weekly using the Psychiatric Patient Nursing Observation Scale. Predictive models were separately developed using six machine learning algorithms: Regularized Logistic Regression (LR), Support Vector Machine (SVM), Extreme Gradient Boosting (XGBoost), Random Forest (RF), Multi-layer Perceptron (MLP), and K-Nearest Neighbor (KNN). Regularized logistic regression was selected as the final algorithm due to its superior predictive performance, indicated by the highest Area Under the Curve (AUC), in both static baseline and dynamic behavioral models. A hierarchical predictive model was then established using regularized logistic regression separately for static baseline risk and dynamic risk fluctuations, subsequently integrated using a weighted fusion approach.

**Results:**

The integrated hierarchical regularized logistic regression model achieved an optimal performance with an area under the curve (AUC) of 0.8741, surpassing both the static baseline model (AUC = 0.7953) and dynamic model (AUC = 0.8003) alone. Optimal predictive performance was obtained with a fusion parameter (*α*) of 0.37, balancing sensitivity (0.7838), specificity (0.8358), and accuracy (0.8173). Key independent predictors included static factors such as history of violence (odds ratio [OR]=4.638), manic symptoms (OR = 7.801), younger age (OR = 0.966), high-risk command hallucinations (OR = 2.602), and dynamic features like anger expression (OR = 4.649), insomnia (OR = 7.422), and auditory hallucinations (OR = 2.092).

**Conclusion:**

The hierarchical machine learning model integrating clinical history and dynamic nursing observations significantly enhances predictive accuracy for violent behavior in schizophrenia inpatients, providing clinicians with valuable tools for timely risk assessment and personalized preventive interventions.

## 1 Introduction

Schizophrenia is a severe psychiatric disorder characterized by disruptions in perception, emotion, cognition, and behavior. Individuals diagnosed with schizophrenia exhibit an elevated risk of violent behaviors (1, 2). Aggressive behaviors, defined as actions with hostile intent, ranging from verbal threats to physical violence against others, occur frequently among hospitalized schizophrenia patients, with reported prevalence rates ranging from 15% to over 50% across different clinical settings (3, 4). Such violence profoundly affects the safety and psychological well-being of other patients and medical staff, significantly increasing the utilization of coercive measures such as restraints and seclusion, which in turn elevate healthcare costs and burden families and institutions (5, 6).

Given these challenges, accurate identification and timely prediction of patients at risk for violent episodes have become crucial objectives in clinical psychiatry. Previous studies have consistently identified static clinical characteristics such as younger age, male gender, history of substance abuse, and prior violent behavior as significant predictors of violence in schizophrenia (7, 8). Additionally, certain dynamic factors, including the presence of command hallucinations, anger expression, and acute psychotic or affective symptoms, have also demonstrated strong associations with violent outcomes (8, 9). Nevertheless, relying solely on static or dynamic factors in isolation often results in limited predictive accuracy, as violent behavior typically arises from the complex interplay between persistent risk markers and acute symptomatic exacerbations (10). This recognition of the interplay between static and dynamic factors has long guided clinical practice and led to the development of structured professional judgment (SPJ) instruments. The most prominent of these is the Historical-Clinical-Risk Management-20 (HCR-20), which provides a standardized framework for clinicians to systematically consider a range of historical (static) and current clinical (dynamic) risk factors (11). While these tools have proven invaluable for structuring the risk assessment process, their primary contribution lies in guiding data collection rather than automating prediction. The final risk estimation still heavily relies on a clinician’s ability to manually and subjectively integrate the scores of a predefined set of variables. This manual integration process may struggle to consistently and objectively weigh the complex, non-linear relationships among a large number of factors, especially when dealing with fine-grained dynamic behavioral data. Therefore, a gap remains for developing more objective, data-driven approaches that can synthesize a richer set of clinical information to enhance predictive accuracy.

Machine learning (ML), with its ability to identify complex, non-linear relationships in large datasets, offers powerful new approaches for psychiatry (12), particularly for challenging tasks like violence prediction. Recent studies leveraging ML techniques have demonstrated superior predictive performance compared to traditional structured assessments, often achieving higher sensitivity and specificity by integrating diverse data types, including clinical histories, nursing observations, and even neuroimaging data (13, 14). However, despite these advancements, there remains substantial room for improvement, especially in models that systematically integrate long-term (static) risk factors and short-term (dynamic) indicators of imminent violent behavior. Nevertheless, most published ML models still adopt flat (mixed) architectures that treat static and dynamic predictors at the same level. Tools such as FOxWeb (15) exemplify this strategy and illustrate a key limitation: within a single-tier framework, subtle fluctuations in modifiable risk factors are often overshadowed by high-impact static correlates (e.g., sex, prior violence), yielding limited incremental predictive value (16). These findings underscore the need for hierarchical or time-aware modeling schemes that can explicitly preserve and optimally weight the distinct contributions of baseline vulnerability and acute state-dependent risk.

To address this gap, this study develops and validates a novel hierarchical machine learning model designed to explicitly separate and optimally integrate static and dynamic risk factors. In contrast to the ‘flat’ architectures previously discussed, our approach employs a two-stage process. First, a baseline risk model is trained exclusively on static clinical history data to establish each patient’s long-term, underlying vulnerability. Concurrently, a separate dynamic risk model is trained on weekly nursing observation data to capture the short-term fluctuations in symptoms and behaviors that may signal imminent violent episodes. In the final stage, the predictions from these two specialized models are integrated via a weighted fusion strategy to generate a single, comprehensive risk score. The principal advantage of this hierarchical design is its ability to prevent the powerful, stable signals from static predictors from diluting or overshadowing the more subtle, yet clinically critical, dynamic indicators. By compelling the model to learn from each data stream independently before integration, our method aims not only to enhance overall predictive accuracy but also to provide a more interpretable framework that clearly distinguishes the contributions of a patient’s stable predispositions versus their acute clinical state.

The main contributions of this paper can be summarized as follows: (1) We construct a robust foundation for model development by building a rich clinical dataset that integrates static clinical data with high-granularity dynamic indicators from weekly nursing observation scales. (2) We propose a novel hierarchical machine learning framework that structurally disentangles static and dynamic risk factors to overcome the “overshadowing” limitations of traditional ‘flat’ models. (3) As a result, we demonstrate significantly enhanced predictive accuracy and provide a more clinically interpretable tool that clarifies the source of patient risk, thereby enabling more targeted and personalized preventive strategies.

## 2 Materials and methods

This section systematically details the methodology used to develop and evaluate a hierarchical predictive model for violent behavior in schizophrenia patients. To ensure clarity and reproducibility, we present our approach in a logical sequence. We begin by describing the study population, including inclusion/exclusion criteria and baseline characteristics. Following this, we detail the data collection process, distinguishing between static clinical history and dynamic nursing observation features. We then provide a precise operational definition for patient grouping based on documented violent behavior. Subsequently, we outline the critical data preprocessing steps, including data cleaning, normalization, and the strict partitioning of data into training and independent test sets. The core of this section describes the construction of our hierarchical model, covering the development of baseline statistical models and machine learning models, hyperparameter optimization via cross-validation, and the model fusion strategy. Finally, we specify the evaluation metrics and the framework used to assess the final model’s performance on the unseen test data, ensuring an objective measure of its generalizability.

### 2.1 Study population

#### 2.1.1 Study subjects and inclusion/exclusion criteria

In this retrospective study, we reviewed the electronic medical records of patients admitted to a psychiatric hospital in Liaoning Province between July 2021 and July 2024. The inclusion criteria for participants were as follows: (1) met the diagnostic criteria for schizophrenia according to the International Classification of Diseases, 10th Revision (ICD-10); (2) were aged between 18 and 65 years; (3) had a total hospitalization duration of greater than 2 weeks; and (4) had a cumulative total of at least 2 completed assessments from the Psychiatric Patient Nursing Observation Scale, which was administered weekly. The primary exclusion criterion was the presence of severe or unstable physical illnesses or organic brain disorders that could confound the assessment of violent behavior. To ensure that dynamic behavioral data reflected pre-incident behavior, we also excluded patients whose first violent episode occurred during the initial week of hospitalization, as these patients lacked sufficient pre-event observation data for meaningful analysis. Initially, a total of 360 patients were identified. After applying the inclusion and exclusion criteria, 14 patients were removed. This resulted in a final sample of 346 participants for analysis, yielding an effective rate of 96.11%.

#### 2.1.2 Demographic and clinical baseline characteristics

The analysis was based on a final cohort of 346 patients. The mean age of the participants was 43.8 ± 12.5 years (range: 18–65 years), with 190 (54.9%) being male and 156 (45.1%) being female. Regarding their sociodemographic and clinical background, a majority of patients were unmarried (75.1%), had an educational level of junior high school or below (76.3%), and were employed (81.2%). The median duration of illness was 14 years (interquartile range: 10–23 years), and the mean duration of hospitalization was 6.34 ± 2.67 weeks. A history of substance abuse was recorded in 34.4% of patients, while a prior history of violence was documented in 75.4%.

### 2.2 Data collection

#### 2.2.1 Data sources and composition

Data for this study were systematically compiled from two primary sources: the hospital’s Electronic Medical Record (EMR) system and the Psychiatric Patient Nursing Observation Scale. These sources provided two distinct types of data: static features, representing baseline clinical history extracted once upon admission, and dynamic features, reflecting behavioral and symptomatic changes collected weekly throughout the patient’s hospitalization. The subsequent subsections provide a detailed account of the variables collected from each source.

#### 2.2.2 Static features: clinical history

A total of 18 demographic and baseline clinical characteristics were retrospectively extracted from patients’ electronic medical records upon admission. These static features included age, gender, occupation, duration of illness, marital status, educational level, personality traits, history of substance abuse, prior incidents of violence, high-risk command hallucinations, persecutory delusions, disturbances in thought processes, abnormalities in sensory perception, intelligence assessments, attention deficits, memory impairment, depressive or hopeless feelings, and manic symptoms.

These features were selected due to their documented associations with violence risk in psychiatric settings. Demographic factors such as younger age and male sex have been linked to a higher propensity for aggressive behavior in clinical populations (17, 18). Socio-economic variables (occupation, marital status, education level) were included because unstable employment, lack of social support (e.g., being unmarried), or lower educational attainment can contribute to stress and diminished coping, which may in turn increase the likelihood of violent incidents (18). Patients’ long-standing personality traits were considered as well, since certain trait profiles (especially those involving impulsivity or antisocial features) are known to predispose individuals to violent behavior (18). A history of substance abuse and prior violent acts are among the most robust predictors of future violence; numerous studies have found that co-morbid substance use can dramatically elevate violence risk, and past violent behavior is the single strongest predictor of subsequent aggression (1, 19). Specific psychopathological symptoms at admission — notably, persecutory delusions and command auditory hallucinations directing the patient to harm others — have been shown to significantly heighten the risk of violent behavior, underlining the importance of assessing these high-risk symptoms (18, 20). Likewise, disturbances in thought processes and other abnormalities in sensory perception (hallucinations) indicate acute psychotic disorganization, which can impair judgment and self-control. Cognitive deficits, as evidenced by low performance on intelligence tests or observed attention and memory impairments, may further contribute to violence risk by limiting the patient’s impulse control and problem-solving abilities (1). Finally, extreme mood symptoms were included because affective states can modulate aggression: for instance, manic symptoms are frequently accompanied by heightened irritability and disinhibition, factors which correlate with increased aggression potential (17). Incorporating these static factors provides a comprehensive baseline profile, grounded in prior research, to inform violence risk assessments in psychiatric inpatients.

#### 2.2.3 Dynamic features: nursing observation scale

Dynamic behavioral data were collected on a weekly basis using the *Psychiatric Patient Nursing Observation Scale*, a structured nurse-rated instrument derived from the validated Nurses’ Observation Scale for Inpatient Evaluation (NOSIE-30) (21). The NOSIE-30 is a validated 30-item scale originally developed by Honigfeld and colleagues for quantifying the behavior of psychiatric inpatient. It has been widely used in clinical settings and demonstrated robust psychometric properties across cultures, with studies reporting high internal consistency (Cronbach’s *α* ≈ 0.80–0.90) and strong inter-rater reliability (22). In our study, this scale was expanded to 39 items by incorporating additional clinically relevant observations (such as the patient’s insight into illness and expressed desire for discharge) to enhance its comprehensiveness. Each item was rated on a 4-point Likert scale from 0 (normal or not present) to 3 (severe or most abnormal), reflecting the frequency and intensity of that behavior during the observation period.

Assessment Procedure: All inpatients were evaluated with this scale at the end of each week of hospitalization. Specifically, every Friday afternoon the primary nurse for each patient completed the 39-item rating, based on their observations of the patient’s behavior and symptoms over the preceding 7 days (from Saturday through Friday).

Scale Composition: These features included adherence to ward regulations; ability to manage personal belongings such as clothes and snacks; participation in bed-making, cleaning, occupational therapy, recreational activities, and broadcast gymnastics; appropriate dressing according to ambient temperature; self-report of physical discomfort; interpersonal interactions and attitudes toward family, peers, and staff; emotional expressions including responses to humor; personal hygiene practices (face washing, teeth brushing, foot washing, grooming hair, sanitation regarding toileting and menstruation); dietary habits; episodes of anger, agitation, increased or rapid speech, self-talk, inappropriate laughter; presence of auditory hallucinations; sedentary or inactive behaviors such as prolonged sitting or lying down; psychomotor slowing; insomnia; episodes of crying; subjective feelings of depression; negative self-evaluation; insight into illness; and expressed desire for discharge.

### 2.3 Patient grouping

#### 2.3.1 Operational definition of violent behavior

In this study, “violent behavior” was operationally defined as any intentional use of physical force or power, whether threatened (e.g., raising a fist, adopting an aggressive posture) or actual, against another person or property during the patient’s hospitalization, which either results in or has a high likelihood of resulting in injury, psychological harm, or property damage.

This definition was designed to align with the broad definition of violence by the World Health Organization (WHO) in the *Global Report on Violence and Health* and is informed by the operational criteria for “physical aggression” found in commonly used psychiatric risk assessment tools, such as the HCR-20, the Overt Aggression Scale (OAS), and the Brøset Violence Checklist (BVC).

Included Behaviors: Examples of behaviors meeting this definition included, but were not limited to: beating, punching, kicking, pushing, grabbing, biting, throwing objects at others, or using an object as a weapon to attack another person, as well as destructive acts toward property, such as intentionally smashing windows or breaking doors.

Excluded Behaviors: To ensure the specificity of the classification, purely verbal threats, insults, or non-aggressive defiant behaviors (e.g., refusal of medication) were not classified as violent behavior. Similarly, acts of self-harm or suicidality were explicitly excluded, unless they escalated into or occurred concurrently with physical aggression directed toward others.

#### 2.3.2 Grouping process and criteria

A rigorous assessment and grouping protocol was established to accurately assign patients to the “violent group” and “non-violent group”:

1. Raters and Training: Two attending psychiatrists, trained and familiar with violence risk assessment standards, served as raters. Their training included an in-depth review of the operational definition used in this study and discussions of case vignettes to ensure consistency in applying the criteria.
2. Data Retrieval: The two raters independently and retrospectively reviewed the complete electronic medical records (EMRs), nursing shift notes, and adverse event reports for each patient’s entire hospitalization period. The review focused on narrative text describing patient behavior, supplemented by keyword searches (e.g., “hit”, “attack”, “assault”, “destroy”, “smash”) to screen for potential incidents.
3. Event Tagging and Consistency Check: Raters independently tagged any behavioral incidents identified according to the definition in Section 2.3.1. After the evaluation of all cases was complete, the tagged results were compared. Any disagreements between the raters were resolved by a joint review of the original records and discussion to reach a consensus.
4. Final Grouping: Based on the final consensus, any patient with at least one documented incident (≥ 1) meeting the criteria for violent behavior during their hospitalization was classified into the violent group (n = 123). Patients with no such documented incidents were classified into the non-violent group (n = 223). Detailed distributions of age, duration of illness, gender, marital status, occupation, educational level, substance abuse history, and history of violence between the violent and non-violent groups are presented in **Table 1**.

**Table 1:** Distribution of patient characteristics in violent and non-violent behavior groups.

Characteristics	Category or unit	Violent group (n=123)	Non-violent group (n=223)	Total (n=346)
Age	Years	38.80 ± 11.40	46.50 ± 12.20	43.80 ± 12.50
Duration of illness	Years	13 (8, 20)	16 (11, 24)	14 (10, 23)
Gender	Male	72 (58.50%)	118 (52.90%)	190 (54.90%)
	Female	51 (41.50%)	105 (47.10%)	156 (45.10%)
Marital Status	Married	26 (21.10%)	60 (26.90%)	86 (24.90%)
	Unmarried	97 (78.90%)	163 (73.10%)	260 (75.10%)
Occupation	Employed	91 (74.00%)	190 (85.20%)	281 (81.20%)
	Unemployed	32 (26.00%)	33 (14.80%)	65 (18.80%)
Educational Level	High School or above	40 (32.50%)	42 (18.80%)	82 (23.70%)
	Junior High or below	83 (67.50%)	181 (81.20%)	264 (76.30%)
Substance Abuse History	Yes	40 (32.50%)	79 (35.40%)	119 (34.40%)
	No	83 (67.50%)	144 (64.60%)	227 (65.60%)
Violence History	Yes	110 (89.40%)	151 (67.70%)	261 (75.40%)
	No	13 (10.60%)	72 (32.30%)	85 (24.60%)

### 2.4 Data preprocessing

#### 2.4.1 Dataset partitioning

Before any preprocessing, we performed a single random split of the entire sample (N = 346) and consistently used this partitioning in all subsequent experiments. Specifically, patients were randomly assigned by patient ID such that 70% were allocated to the training set and the remaining 30% to an independent test set. The training set was exclusively dedicated to model development, encompassing feature selection, model training, and hyperparameter tuning; the test set remained completely isolated during the development process and was only used once, after the model was finalized, to objectively assess its performance on unseen data. This fixed partitioning strategy helps prevent data leakage and ensures the reliability and reproducibility of the evaluation results.

#### 2.4.2 Data cleaning

Prior to analysis, the dataset was cleaned to ensure accuracy and completeness. Records with missing key information or insufficient hospitalization duration were excluded during initial screening. For remaining data, necessary format transformations were applied—such as extracting numeric values from free-text entries for “duration of illness.” All fields were checked for outliers, and residual missing values were imputed using the median for continuous variables and the mode for categorical variables, minimizing bias while preserving data integrity.

#### 2.4.3 Data encoding

Categorical variables were encoded into numeric representations suitable for machine learning algorithms. Binary attributes (such as sex, marital status, employment status, presence of substance abuse history, prior violence history, and key psychotic symptoms like command hallucinations or persecutory delusions) were converted into binary indicator variables (0 = absence, 1 = presence). This binary encoding ensures that these features are treated as dichotomous flags in the models. For ordinal variables, we preserved their rank order by mapping categories to integer codes. Notably, education level was encoded on an increasing scale (for example, 0 for no formal schooling or elementary school, 1 for junior high school, 2 for high school or vocational training, and 3 for college degree or higher), reflecting higher educational attainment with larger numeric values. The personality trait variable (introverted vs. extroverted) was similarly coded as a binary feature. These encoding steps were applied after careful verification of category values in the dataset (with any unexpected or out-of-vocabulary entries logged and reviewed). The dynamic nursing observation features were already numeric (ordinal ratings from 0 to 3 for each behavioral item) and thus were directly usable without additional encoding, aside from ensuring that they were read as numerical data types.

#### 2.4.4 Normalization

After encoding, all continuous numerical features were normalized to ensure they were on comparable scales, a step that prevents features with larger ranges from dominating others in distance-based or gradient based modeling algorithms. In practice, we applied standard z-score normalization to the relevant features. Each continuous feature (such as age, illness duration, and each of the weekly observation scale item scores) was rescaled by subtracting the mean and dividing by the standard deviation of that feature (computed from the training set data). This transformation yielded features with a mean of 0 and a standard deviation of 1.

### 2.5 Model construction

#### 2.5.1 Prediction task definition

The goal of this study is to identify which inpatients with schizophrenia are likely to exhibit violent behavior during hospitalization. This is formulated as a binary classification problem with labels defined as 1 for violent patients and 0 for non-violent patients.

We construct two separate models to estimate this risk based on different sources of information, and then combine their outputs:

- Static risk model. The inputs to this model are 18 baseline clinical features collected at admission, including demographic variables (e.g. age, sex, marital status) and clinical history (e.g. previous violence, substance use, personality traits). This model outputs a probability, denoted by $R_{0}$ , that the patient belongs to the violent group. It captures long-term, stable risk factors.
- Dynamic behavioral model. The inputs are 39 behavioral features from the Nursing Observation Scale. For each patient, we average all weekly scores prior to the first violent incident (for violent patients) or all weekly scores during hospitalization (for non-violent patients) to obtain a single 39-dimensional vector. Using this vector, the model outputs a probability, denoted by $R_{\text{dyn}}$ , that the patient belongs to the violent group. This model focuses on the patient’s overall behavioral state.

To integrate these two risk estimates, we compute a weighted combination


$$R^{*}=\alpha R_{0}+(1-\alpha )\;R_{\text{dyn}},$$

where *α* is a weight between 0 and 1 chosen on the training set. A decision threshold is applied to *R* ^∗^ to convert it into a final binary prediction.

We adopt a hierarchical model design, which not only addresses the complexity of the data structure but also simulates the clinical thought process used by physicians when assessing risk. Specifically, in clinical practice physicians tend to rely on two types of information: first, long-term or static data about the patient—such as age, past medical history, and family background—forming a baseline risk assessment; and second, recent dynamic information—such as behavioral manifestations, emotional states, and daily symptoms over the past few weeks—which is used to adjust the baseline and derive the current risk. This mode of thinking helps physicians integrate long-term risk with recent changes to achieve a more comprehensive and timely assessment. Thus, the hierarchical model aligns the data with this separation of “long-term versus short-term” information and matches the clinical reasoning process, making the model structure easier to understand and trust, and thereby better supporting clinical decision-making.

#### 2.5.2 Baseline logistic regression model

In order to establish an interpretable benchmark for violent-risk prediction, we first employed a logistic regression (LR) model. Logistic regression is a classical supervised learning algorithm for binary classification: it maps a linear combination of the input variables onto a probability in the interval [0,1], thereby producing the likelihood that the instance belongs to a particular class. The model assumes that the independent variables are linearly related to the log–odds of the outcome and uses an S–shaped sigmoid function to map the linear predictor to a probability (23). Because of its simplicity and the ease with which coefficients can be interpreted, logistic regression is well suited to provide a transparent baseline against which more complex models can later be compared (24).

Step 1: Univariate analysis.

To identify potential risk factors associated with violent behavior, each candidate feature underwent univariate statistical analysis prior to modeling. The purpose of this step was to screen variables that exhibited significant differences between the two patient groups (violent versus non-violent) and to provide candidate predictors for subsequent multivariate modeling. All statistical analyses were performed in a Python 3.10 environment with the pandas, NumPy, SciPy and statsmodels libraries. The procedure was as follows:

1. Normality testing. The Kolmogorov–Smirnov test was used to assess whether continuous variables followed a normal distribution. Variables that were normally distributed and homoscedastic were compared between groups using independent–sample *t* tests; variables that violated these assumptions were compared using the Wilcoxon rank–sum test. Categorical variables were compared with chi-square tests or Fisher’s exact test.
2. Univariate regression. To further evaluate the association of each feature with violent behavior, a univariate logistic regression model was fitted on the training set for each static and dynamic feature. Odds ratios (ORs) with 95% confidence intervals (CIs) were calculated, and features with *P <* 0.05 were considered significantly associated with violent behavior and were entered into the subsequent multivariate analysis.

Step 2: Multivariate logistic regression analysis.

Because univariate results may be confounded by covariates, multivariate logistic regression was used to control potential confounders and to identify independent predictors. We employed the Logit function from the statsmodels library to fit multivariate logistic regression models on the training set. The independent variables comprised those that were significant in the univariate analysis, and the dependent variable was the violent-behavior label (1 = violent, 0 = non-violent). Exponentiating the estimated regression coefficients yielded ORs with 95% confidence intervals, reflecting each factor’s independent contribution to violent risk after adjusting for other variables. Statistical significance was defined as *P <* 0.05.

Step 3: Hierarchical model development.

After significant variables were identified, regularized logistic regression models were used to build separate static and dynamic submodels, and these were combined via a fusion strategy to form the final hierarchical predictive model. Throughout model development, patient-level splitting into training and test sets was strictly observed: the training set was used for model training and hyperparameter tuning, and the test set was reserved solely for final performance evaluation.

1. Static baseline model (*R*_0_). The static clinical features selected from Steps 1 and 2 were used as inputs to a regularized logistic regression model for violent-risk prediction. Because regularized logistic regression has tunable regularization parameters, a hyperparameter search was required to identify the optimal combination. Five–fold cross validation combined with grid search was executed on the training set to explore the following parameters (25):

- *Penalty type* (penalty): {l1, l2, elasticnet}. L1 regularization can produce sparse solutions and perform feature selection, whereas L2 regularization shrinks all coefficients but does not drive them exactly to zero.
- *Regularization strength* (C): *C* is the inverse of the regularization parameter; smaller values correspond to stronger regularization. Candidate values were taken on a logarithmic scale from 10^−4^ to 10^4^.
- *Solver* (solver): different optimization algorithms (liblinear, lbfgs, saga) were considered because they perform differently on small versus large data sets and with different regularization schemes (23).
- *Maximum iterations* (maxiter): to ensure convergence, maximum iteration limits of 100 and 200 were tested.

Grid search enumerated all combinations of these parameters; for each combination, five–fold cross validation was used to compute the mean area under the receiver–operating-characteristic curve (AUC) and accuracy. The parameter set with the highest average performance was selected, and the regularized logistic regression model was then retrained on the entire training set with these optimal parameters to produce a static baseline risk score *R*_0_. For interpretability, regression coefficients and ORs for each significant feature were reported.

2. Dynamic behavior model (*R*_dyn_). The same regularized logistic regression framework was applied to the 39 dynamic behavior features that were significant in the previous steps. Because the dynamic features were assessed weekly, each patient’s weekly scores before their first violent incident (for the violent group) or before discharge (for the non-violent group) were averaged to create a single feature vector representing their overall behavioral state. The same grid–search procedure described above was used on the training set to tune the penalty, *C*, solver and maximum iterations, and the optimal model was retrained on the full training set to yield a dynamic-risk score *R*_dyn_ (25).

3. Model fusion and integration. We intentionally used a two-step scheme to combine the static and dynamic sub-models. First, the static and dynamic models were tuned independently to estimate the baseline risk *R*_0_ and the state-dependent risk *R*_dyn_; then a single fusion weight *α* was cross-validated on the training data. This two-step design has three practical motivations: (i) *interpretability* — separate training yields stable, reusable estimates of *R*_0_ and *R*_dyn_, and *α* is a simple, clinically meaningful knob to discuss their relative contributions; (ii) *lower overfitting risk* — a single “giant grid” over both sub-models’ hyperparameters plus *α* would greatly expand the search space on a small dataset, making the selection step itself easy to overfit; the two-step procedure decouples model learning from strategy fusion and keeps the search compact and auditable; and (iii) *modularity and operational reuse* — in practice, hospitals may wish to upgrade or swap either component (for example, adding new nursing indicators) without retraining everything; with late fusion, we can update a sub-model and only re-tune *α*. To integrate both sources we adopted a linear weighted fusion:


$$R=\alpha R_{0}+(1-\alpha )\;R_{\text{dyn}},$$

where α∈[0,1] controls the relative weight of the two components. To determine the optimal *α*, grid search was performed on the training set: values between 0 and 1 were tested in increments of 0.05, and the average AUC over five-fold cross-validation was computed for each candidate. The value yielding the highest mean AUC was selected. The fused score *R* represents the predicted probability of violent behavior. Finally, *R* was compared with a decision threshold (default 0.5); patients with *R* above the threshold were predicted to be violent, and those below were predicted to be non-violent.

#### 2.5.3 Machine learning model construction

To provide strong comparators of model performance and capture complex nonlinear relationships, this study introduces multiple flexible machine learning algorithms beyond the baseline logistic regression. The static model uses all 18 admission baseline features, whereas the dynamic model is based on all 39 behavioral features. We develop and tune these models on the training set and evaluate their generalization performance on an independent test set, in order to assess how well each algorithm discriminates between violent and non-violent patients and to compare the incremental value of dynamic information.

Step 1: Static model development.

For the static features, we trained five classifiers individually: multi-layer perceptron (MLP), random forest (RF), *k*-nearest neighbors (KNN), support vector machine (SVM) and extreme gradient boosting (XGBoost). Training adhered strictly to a training/test split, and hyperparameters were selected on the training set using fivefold cross-validation with grid search or random search. The main tuning points were as follows:

- MLP. The number of hidden layers and neurons, activation functions (e.g. ReLU or tanh), the L2 regularization coefficient and the learning rate were adjusted. Because MLPs are sensitive to feature scaling and require non-convex optimization, cross-validation helps identify a stable network architecture (26).
- Random forest. We tuned the number of trees (n_estimators), the number of features considered at each split (max_features) and the maximum tree depth (max_depth). Minimum sample requirements for splitting nodes (min_samples_split) and for leaf nodes (min_samples_leaf) were also adjusted to control overfitting (27).
- KNN. The key hyperparameter is the number of neighbors *k*. Too small a value leads to overfitting, while too large a value may miss local structure. We searched *k* from 3 to 20, considered different weighting schemes (uniform or distance) and tried Euclidean and Manhattan distance metrics (28).
- SVM. Using a radial basis function (RBF) kernel, we tuned the penalty parameter *C* and the kernel width *γ* via grid search to balance margin maximization and classification error (29).
- XGBoost. We focused on tree depth, minimum child weight and minimum child sample number, subsample and column-subsample ratios, learning rate and the number of boosting rounds. When necessary, additional regularization parameters such as gamma, reg_alpha and reg_lambda were adjusted to reduce overfitting (30).

Once the best parameters were identified via cross-validation on the training set, each model was retrained on the full training data and evaluated on the test set.

Step 2: Dynamic behavioral model development.

The dynamic models followed the same procedure as the static models but used the 39 behavioral features as input. Each classifier (MLP, RF, KNN, SVM, XGBoost) was trained and tuned on the training set and evaluated on the test set. Because the dynamic features capture behavioral changes during hospitalization, models capable of learning complex nonlinear patterns are particularly useful here.

Step 3: Model fusion and integration.

To combine the predictions of the static and dynamic models, we adopted a linear weighted fusion. Let *R*_0_ denote the probability output from the static model and *R*_dyn_ denote the probability from the dynamic model. A fused probability was computed as


$$R=\alpha \;R_{0}+(1-\alpha )\;R_{\text{dyn}},$$

where α∈[0,1] controls the relative contribution of each part. We searched *α* from 0 to 1 in increments of 0.05 using fivefold cross-validation on the training set and selected the *α* with the highest average AUC. Finally, we compared *R* against a decision threshold (default 0.5) on the test set to obtain the final predictions.

### 2.6 Evaluation metrics

To systematically evaluate the clinical value of our hierarchical predictive model, we selected several evaluation metrics that capture both discriminative ability and calibration. Sensitivity (recall), specificity, positive predictive value (PPV), negative predictive value (NPV), accuracy, the area under the receiver–operating-characteristic curve (AUC) and the confusion matrix were computed. These metrics provide complementary information about how well the model distinguishes between violent and non-violent inpatients and how well its predicted probabilities align with observed outcomes.

#### 2.6.1 Sensitivity (recall)

Sensitivity quantifies the proportion of violent patients who are correctly classified as high risk. In violence prediction, missing a patient who subsequently becomes violent (a false negative) may have serious consequences for other patients and staff. Ethicists argue that the harm associated with false negatives is generally viewed as more unacceptable than false positives, so high sensitivity is critical to ensure that potential violent incidents are not overlooked (31).

#### 2.6.2 Specificity

Specificity measures the proportion of non-violent patients who are correctly identified as low risk. High specificity reduces false positives and thus protects patients from unnecessary restrictions or interventions. Ogonah et al. note that instruments with high specificity are most suitable for discharge planning and resource allocation because they avoid overestimating risk and preserve patients’ rights (32).

#### 2.6.3 Positive predictive value and negative predictive value

PPV denotes the proportion of individuals judged to be high risk who subsequently commit violence, whereas NPV represents the proportion of those judged to be low risk who do not later commit violence. Fazel et al. emphasize that many risk assessment tools demonstrate relatively low PPV but high NPV, reflecting the low base rate of violence; in their meta-analysis the median PPV was around 41% but NPV exceeded 90% (33). Reporting PPV and NPV therefore informs clinicians how many high-risk predictions translate into actual violent incidents and how confidently low-risk predictions can be used to rule out future violence.

#### 2.6.4 Accuracy

Accuracy is the overall proportion of correct predictions. Although accuracy provides an intuitive summary, it can be misleading in imbalanced data sets where one class predominates. We therefore report accuracy alongside other metrics to provide context.

#### 2.6.5 AUC

The area under the receiver operating characteristic (ROC) curve summarizes the probability that the model will assign a higher risk score to a randomly selected violent patient than to a randomly selected non-violent patient. As Connors and Large discuss, AUC has become the most widely used measure of discrimination because it is independent of a particular cut-off and relatively unaffected by base-rate differences (34). However, AUC measures discrimination only; a model can have a high AUC yet systematically over- or under-estimate absolute risk (32), so it must be interpreted together with calibration measures.

#### 2.6.6 Confusion matrix

The confusion matrix tabulates true positives, false positives, true negatives and false negatives. It provides a transparent overview of the model’s errors and forms the basis for calculating all other metrics. Examining the confusion matrix helps clinicians understand whether a model tends to over-predict or under-predict violent behavior, informing decisions about acceptable trade-offs between sensitivity and specificity.

By reporting both discrimination metrics (sensitivity, specificity, accuracy, AUC) and calibration metrics (PPV, NPV), our evaluation aligns with recommendations that violence risk assessments should consider multiple aspects of performance. Such a comprehensive evaluation helps balance public safety against patients’ rights in clinical decision-making (32).

## 3 Results

This section systematically presents the core findings of our study on violence prediction. We begin by reporting the results of the univariate analysis for both static clinical features and dynamic nursing observation features to initially screen for potential risk factors significantly associated with violent behavior. Building on this, we then present the findings from the multivariate logistic regression analysis, which aims to identify the key variables that independently predict violence risk. Finally, this section provides a detailed evaluation of the predictive performance of the constructed baseline regularized logistic regression model and the hierarchical machine learning models, comprehensively demonstrating their accuracy, discriminative ability, and clinical value using a range of metrics on an independent test set.

### 3.1 Univariate analysis

To identify the key variables for constructing the predictive models, we first conducted univariate analysis to screen for potential risk factors associated with violent behavior.

#### 3.1.1 Single-factor analysis of static features

Static baseline features were retrospectively extracted from electronic medical records and clinical baseline assessments upon patient admission. Statistical analyses were conducted to evaluate differences between violent and non-violent behavior groups. Continuous variables, such as age and duration of illness, were analyzed using the Mann-Whitney U test due to non-normal distributions. Categorical variables, including binary and ordinal variables, were assessed with the Chi-square (*χ* ^2^) test or Fisher’s exact test, as appropriate. As shown in **Table 2**, several static features demonstrated statistically significant differences (*P <* 0.05) between the two groups. Patients exhibiting violent behaviors were significantly younger (*P <* 0.001) and had a shorter disease duration (*P* = 0.003). A history of violence was also more prevalent in the violent group (*P <* 0.001). Additionally, the violent group showed higher incidences of mania (*P* = 0.002), hopelessness or depression (*P* = 0.011), and high-risk command hallucinations (*P* = 0.015). Significant differences were also observed regarding education level (*P* = 0.003) and occupation status (*P* = 0.016).

**Table 2:** Single-factor analysis of significant static features between violent and non-violent groups.

Feature	Feature type	Test method	P	Significant
Age	Continuous variable	Mann-Whitney U test	*<*0.001	Yes
Violence History	Binary variable	Chi-square test	*<*0.001	Yes
Mania	Binary variable	Fisher’s exact test	0.002	Yes
Disease Duration	Continuous variable	Mann-Whitney U test	0.003	Yes
Education Level	Ordinal categorical variable	Mann-Whitney U test	0.003	Yes
Hopelessness/Depression	Binary variable	Chi-square test	0.011	Yes
High-risk Command Hallucinations	Binary variable	Chi-square test	0.015	Yes
Occupation	Binary variable	Chi-square test	0.016	Yes

#### 3.1.2 Single-factor analysis of dynamic features

Dynamic behavioral features assessed weekly using the Psychiatric Patient Nursing Observation Scale were analyzed to determine differences between the violent and non-violent patient groups. Each of the 39 dynamic variables was rated on an ordinal scale from 0 (absent or normal) to 3 (severe or highly abnormal). The Mann-Whitney U test or Chi-square test was employed for statistical comparison, depending on the distribution characteristics of each variable. Significant differences (*P <* 0.05) were observed in 31 of the 39 dynamic variables. The detailed statistical results are summarized in **Table 3**. Patients in the violent behavior group consistently exhibited significantly higher severity in behavioral disturbances such as anger expression (*P <* 0.001), lower adherence to ward regulations (*P <* 0.001), reduced cooperation with staff (*P <* 0.001), increased auditory hallucinations (*P <* 0.001), and greater difficulty participating in structured activities like exercises (*P <* 0.001), recreational activities (*P <* 0.001), and occupational therapy (*P <* 0.001). Moreover, significant impairments in basic daily activities such as cleaning (*P <* 0.001), personal affairs management (*P <* 0.001), face washing (*P <* 0.001), teeth brushing (*P <* 0.001), and maintaining neat appearance (*P <* 0.001) were more prevalent among violent patients. Additional notable behavioral differences included higher rates of insomnia (*P <* 0.001), agitation (*P <* 0.001), inappropriate laughter (*P* = 0.002), and self-talking behaviors (*P <* 0.001). Violent patients also demonstrated significantly decreased engagement in interpersonal and social interactions, such as reduced conversation with others (*P* = 0.008), less interest in surroundings (*P <* 0.001), and diminished family concern (*P* = 0.001). These findings underscore the predictive utility of dynamic behavioral monitoring in identifying patients at higher risk for violent behaviors during hospitalization.

**Table 3:** Single-factor analysis of significant dynamic features between violent and non-violent groups.

Feature	Feature type	Test method	P	Significant
Anger Expression	Ordinal Variable	Mann-Whitney U test	*<*0.001	Yes
Rule Compliance	Ordinal Variable	Mann-Whitney U test	*<*0.001	Yes
Cooperation with Staff	Ordinal Variable	Mann-Whitney U test	*<*0.001	Yes
Auditory Hallucinations	Ordinal Variable	Mann-Whitney U test	*<*0.001	Yes
Exercise Participation	Ordinal Variable	Mann-Whitney U test	*<*0.001	Yes
Recreational Activities	Ordinal Variable	Mann-Whitney U test	*<*0.001	Yes
Insomnia	Ordinal Variable	Mann-Whitney U test	*<*0.001	Yes
Cleaning	Ordinal Variable	Mann-Whitney U test	*<*0.001	Yes
Work Therapy Participation	Ordinal Variable	Mann-Whitney U test	*<*0.001	Yes
Personal Affairs Management	Ordinal Variable	Mann-Whitney U test	*<*0.001	Yes
Interest in Surroundings	Ordinal Variable	Mann-Whitney U test	*<*0.001	Yes
Attitude Toward Others	Ordinal Variable	Mann-Whitney U test	*<*0.001	Yes
Face Washing	Ordinal Variable	Mann-Whitney U test	*<*0.001	Yes
Bed Making	Ordinal Variable	Mann-Whitney U test	*<*0.001	Yes
Teeth Brushing	Ordinal Variable	Mann-Whitney U test	*<*0.001	Yes
Neat Appearance	Ordinal Variable	Mann-Whitney U test	*<*0.001	Yes
Talking to Self	Ordinal Variable	Mann-Whitney U test	*<*0.001	Yes
Illness Awareness	Ordinal Variable	Mann-Whitney U test	*<*0.001	Yes
Hand Washing Before Meals	Ordinal Variable	Mann-Whitney U test	*<*0.001	Yes
Agitation	Ordinal Variable	Mann-Whitney U test	*<*0.001	Yes
Laughs at Jokes	Ordinal Variable	Mann-Whitney U test	*<*0.001	Yes
Psychomotor Retardation	Ordinal Variable	Mann-Whitney U test	*<*0.001	Yes
Family Concern	Ordinal Variable	Mann-Whitney U test	0.001	Yes
Clothing Adjustment	Ordinal Variable	Mann-Whitney U test	0.001	Yes
Discussing Personal Interests	Ordinal Variable	Mann-Whitney U test	0.001	Yes
Inappropriate Laughing	Ordinal Variable	Mann-Whitney U test	0.002	Yes
Rapid Speech	Ordinal Variable	Mann-Whitney U test	0.003	Yes
Foot Washing	Ordinal Variable	Mann-Whitney U test	0.006	Yes
Hair Grooming	Ordinal Variable	Mann-Whitney U test	0.008	Yes
Conversation with Others	Ordinal Variable	Mann-Whitney U test	0.008	Yes
Physical Discomfort Description	Ordinal Variable	Mann-Whitney U test	0.012	Yes

### 3.2 Multivariate logistic regression analysis

Building upon the univariate analysis, we then conducted multivariate logistic regression analysis to further control for confounding factors and identify the core factors that independently predict violent behavior.

#### 3.2.1 Logistic regression analysis of static features

Based on the results from the univariate analysis, static features demonstrating significant differences (*P <* 0.05) between the violent and non-violent patient groups were included in a multivariate logistic regression model to identify independent predictors of violent behavior among schizophrenia patients. Specifically, variables such as age, duration of illness, history of violence, manic symptoms, and other statistically significant factors (a total of eight variables) were initially selected for inclusion.

Multivariate logistic regression analysis identified four static clinical characteristics as independent predictors of violent behavior, as shown in **Table 4**. Patients with a history of violence had significantly increased odds of exhibiting violent behavior (OR = 4.638, 95% CI: 2.169–9.918, *P <* 0.001). Additionally, manic symptoms substantially elevated the risk of violent incidents (OR = 7.801, 95% CI: 1.449–41.997, *P* = 0.017). Younger age was associated with a higher likelihood of violent behaviors (OR = 0.966, 95% CI: 0.937–0.997, *P* = 0.030). Patients experiencing high-risk command hallucinations were also significantly more likely to engage in violent behaviors (OR = 2.602, 95% CI: 1.033–6.553, *P* = 0.043).

**Table 4:** Multivariate logistic regression of static features predicting violent behavior.

Feature	Coefficient	P	OR	95% CI
Violence History	1.534	*<*0.001	4.638	2.169–9.918
Mania	2.054	0.017	7.801	1.449–41.997
Age	-0.034	0.030	0.966	0.937–0.997
High-risk Command Hallucinations	0.956	0.043	2.602	1.033–6.553

These findings suggest that a history of violence, presence of manic symptoms, younger age, and high-risk command hallucinations are independently associated with an increased risk of violent behavior among schizophrenia patients, highlighting their potential utility as predictive indicators for early clinical assessment and preventive interventions.

#### 3.2.2 Logistic regression analysis of dynamic scale features

Based on the results from the univariate analysis, dynamic behavioral variables that showed significant differences ( P<0.05 ) between the violent and non-violent patient groups were included in a multivariate logistic regression model. This analysis aimed to identify dynamic clinical features independently associated with violent behavior among schizophrenia patients. Variables such as anger expression, insomnia, auditory hallucinations, and other statistically significant factors (a total of 33 variables) were initially selected.

Multivariate logistic regression analysis identified five dynamic behavioral features independently predictive of violent behaviors, as shown in **Table 5**. Specifically, higher ratings of anger expression significantly increased the likelihood of violent incidents (OR =4.649 , 95% CI: 2.555 – 8.458 , P<0.001 ). Similarly, insomnia (OR =7.422 , 95% CI: 2.212 – 24.900, P=0.001) and auditory hallucinations (OR =2.092, 95% CI: 1.319 – 3.317,  P=0.002) were also significantly associated with increased risk. Conversely, higher ratings of psychomotor retardation (OR =0.467 , 95% CI: 0.235 – 0.927, P=0.030 ) and greater illness awareness (OR =0.636 , 95% CI: 0.415 – 0.973 , P=0.037 ) were associated with reduced odds of violent behavior.

**Table 5:** Multivariate logistic regression of dynamic features predicting violent behavior.

Feature	Coefficient	P-value	OR	95% CI
Anger Expression	1.536	*<*0.001	4.649	2.555–8.458
Insomnia	2.004	0.001	7.422	2.212–24.900
Auditory Hallucinations	0.738	0.002	2.092	1.319–3.317
Psychomotor Retardation	-0.762	0.030	0.467	0.235–0.927
Illness Awareness	-0.453	0.037	0.636	0.415–0.973

These results emphasize that dynamic changes in anger, sleep disturbances, and auditory hallucinations independently predict an increased risk of violent episodes. In contrast, psychomotor slowing and better illness insight appear to confer protective effects. Regular monitoring of dynamic factors, such as anger expression, insomnia, and auditory hallucinations, facilitates the early identification of violence risk in patients with schizophrenia and the prompt implementation of preventive measures.

### 3.3 Performance of hierarchical machine learning models

To systematically verify the effectiveness of the hierarchical fusion strategy, we adopted a step-wise modeling and evaluation approach tailored to the feature selection results: the regularized logistic regression model used only the significant features identified through univariate and multivariate analyses, with the static model including history of violence, manic symptoms, age and high-risk command hallucinations, and the dynamic model including anger expression, insomnia, auditory hallucinations, psychomotor retardation and illness awareness. This was intentional: regularized logistic regression assumes a linear relationship between predictors and the log-odds and prefers parsimonious models, so using a small number of significant features avoids overfitting and aids clinical interpretation. In contrast, the other machine learning algorithms (SVM, XGBoost, random forest, MLP and KNN) can capture complex nonlinear relationships and handle highly correlated features, so all 18 baseline variables or all 39 dynamic nursing-observation variables were used as inputs to retain as much information as possible. All models were tuned using five-fold cross-validation on the same training set to ensure fair comparison.

#### 3.3.1 Performance of the static baseline model

For the 18 baseline features at admission, we trained six algorithms. The regularized logistic regression model used the four significant predictors mentioned above and tuned the type of regularization (L1, L2 or Elastic Net), regularization parameter C , solver and number of iterations via grid search. The optimal static LR model employed L2 regularization, C=1.0 , the liblinear solver and 200 iterations; it achieved an AUC of 0.7953, the highest among all static models, and balanced sensitivity (0.4846), specificity (0.8955) and accuracy (0.7500). The other nonlinear models were trained on all 18 baseline features and tuned similarly: the MLP used two hidden layers (with 64 and 32 neurons), a ReLU activation function, a learning rate of 0.001 and an L2 regularization coefficient of 0.001; the random forest used 200 trees, a maximum depth of 6 and sqrt as the maximum feature fraction; KNN used k=7 , distance weighting and the Euclidean distance metric; SVM used an RBF kernel with C=1 and γ=0.01 ; and XGBoost was set with a maximum depth of 4, a learning rate of 0.1, 100 boosting rounds, subsample and column subsample rates of 0.8, and a minimum child weight of 1. Under these optimal settings, regularized logistic regression still exhibited the strongest discrimination, indicating that a carefully selected subset of static features adequately captures long-term risk. Detailed performance metrics and model comparisons are presented in **Table 6**.

**Table 6:** Predictive performance of static baseline models for violent behavior.

Model type	Sensitivity	Specificity	Positive predictive value	Negative predictive value	Accuracy	AUC
MLP	0.4595	0.8806	0.6800	0.7468	0.7308	0.5484
RF	0.2973	0.8060	0.4583	0.6750	0.6250	0.6769
KNN	0.2973	0.8358	0.5000	0.6829	0.6442	0.6480
SVM	0.4865	0.8955	0.7200	0.7595	0.7500	0.7691
LR	0.4846	0.8955	0.7200	0.7595	0.7500	0.7953
XGBoost	0.4865	0.9104	0.7500	0.7625	0.7596	0.7749

#### 3.3.2 Performance of the dynamic behavioral model

Dynamic behavioral models were developed using weekly nursing-observation scores following a similar procedure. The dynamic LR model used only the five significant behavioral indicators and tuned the regularization type, C and solver. The best dynamicLR model adopted L2 regularization, C=0.5 and the liblinear solver; it achieved an AUC of 0.8003, specificity of 0.8507 and positive predictive value of 0.6552, showing that behavioral fluctuations are highly predictive of imminent violence. For comparison, MLP (two hidden layers with 128 and 64 neurons, ReLU activation and a 0.001 learning rate), random forest (300 trees, maximum depth 10, sqrt feature sampling), KNN ( k=9 , distance weighting), SVM (RBF kernel, C=0.5 , γ=0.005 ) and XGBoost (maximum depth 3, learning rate 0.05, 200 boosting rounds, subsample and column subsample rates of 0.8) were all trained using the full set of 39 features, but none exceeded the AUC of LR. This further indicates that the small number of dynamic predictors identified in the univariate and multivariate analyses contain most of the predictive information. Detailed performance metrics and model comparisons are presented in **Table 7**.

**Table 7:** Predictive performance of dynamic behavioral models for violent behavior.

Model type	Sensitivity	Specificity	Positive predictive value	Negative predictive value	Accuracy	AUC
MLP	0.6216	0.7612	0.5897	0.7846	0.7115	0.7370
RF	0.6486	0.7612	0.6000	0.7969	0.7212	0.7475
KNN	0.2703	0.9851	0.9091	0.7097	0.7308	0.6846
SVM	0.5405	0.8209	0.6250	0.7639	0.7212	0.7348
LR	0.5135	0.8507	0.6552	0.7600	0.7308	0.8003
XGBoost	0.4865	0.8060	0.6061	0.7606	0.7115	0.7402

#### 3.3.3 Performance of the integrated model

**Figure 1:**
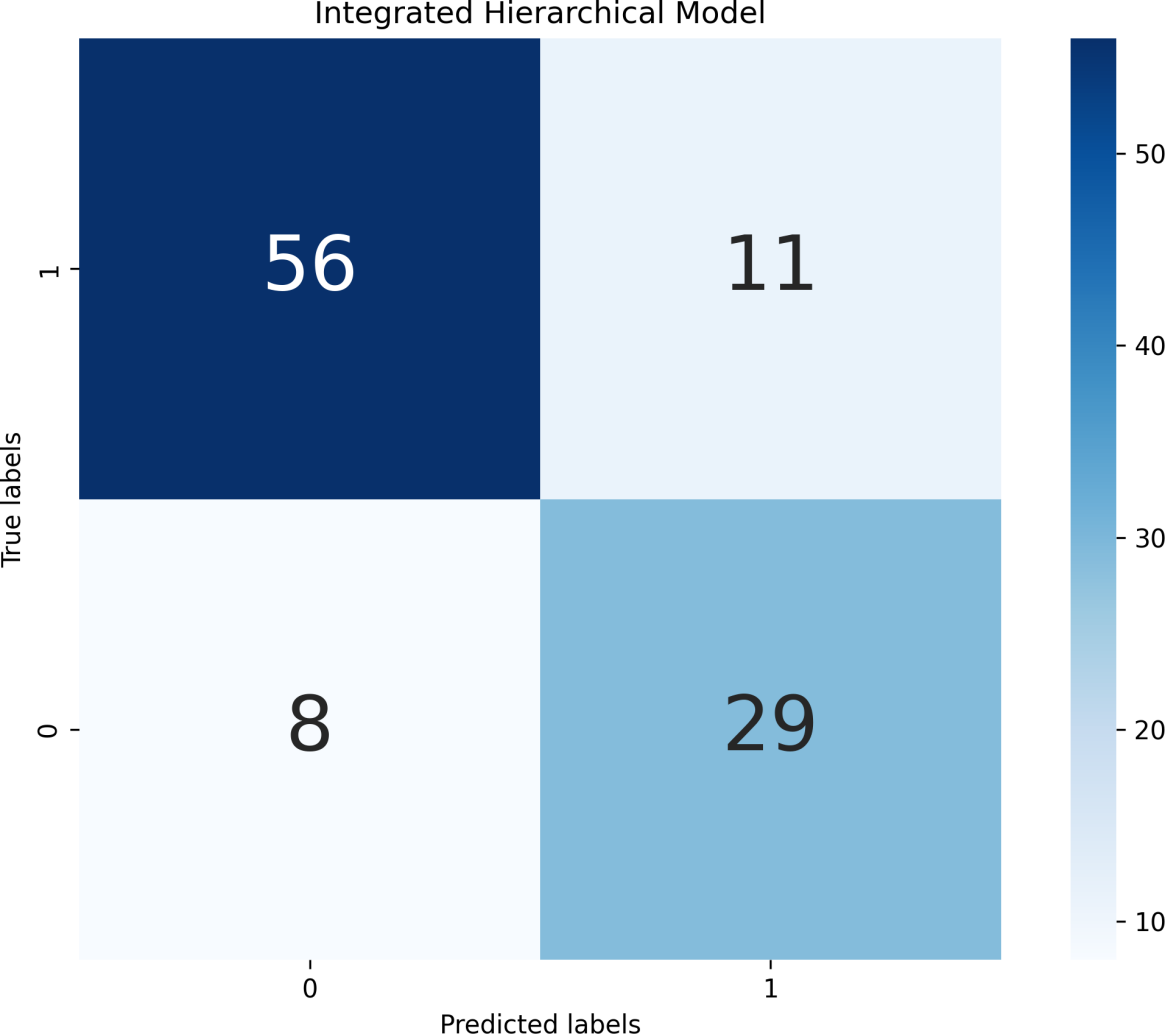
Confusion matrix of integrated hierarchical model predictions.

To integrate long-term and short-term risk information, we combined the static and dynamic predictions using a decision-level weighted fusion, $R=\alpha \;R_{0}+(1-\alpha )\;R_{\text{dyn}}$ . The weight α was selected by grid search on the training set with a step size of 0.05; the optimal α varied by model pair: the LR+LR combination had an optimal weight of 0.37, while the optimal weights for the SVM+SVM, XGBoost+XGBoost, random forest+random forest, MLP+MLP and KNN+KNN combinations were 0.40, 0.35, 0.45, 0.43 and 0.30 respectively. Because LR achieved the highest AUC in both static and dynamic submodels, we ultimately adopted the LR+LR fusion as the hierarchical predictor. This fused model increased the AUC to 0.8741, with sensitivity 0.7838, specificity 0.8358 and accuracy 0.8173, clearly outperforming the individual static or dynamic models. The performance improvement indicates that static predictions provide a stable baseline, dynamic indicators capture short-term exacerbations, and the weighted combination of the two probability outputs yields more accurate and timely predictions of violent behavior in hospitalized schizophrenia patients. Detailed experimental results can be found in **Figure 1** and **Table 8**.

**Table 8:** Predictive performance of the integrated hierarchical model for violent behavior.

Model type	Sensitivity	Specificity	Positive predictive value	Negative predictive value	Accuracy	AUC
LR + LR	0.7838	0.8358	0.7250	0.8750	0.8173	0.8741

## 4 Discussion

The present study developed and validated a hierarchical machine learning model integrating both static clinical characteristics and dynamic behavioral observations to predict violent behavior among hospitalized schizophrenia patients. The key findings underscore the feasibility and efficacy of employing a two-tier predictive framework, achieving significantly higher predictive performance compared to relying solely on static baseline characteristics or dynamic behavioral indicators independently.

Our study identified multiple independent risk factors from static clinical features, specifically history of violence, manic symptoms, younger age, and high-risk command hallucinations, as significant predictors of violent behavior. These findings align closely with existing literature, where prior violence, manic presentations, and younger age have consistently been identified as robust predictors of violent incidents among schizophrenia patients (1, 7, 35, 36). Additionally, the association between high-risk command hallucinations and violent behaviors is also well documented, highlighting how psychotic symptoms can exacerbate impulsivity and aggression (35, 37, 38).

Regarding dynamic behavioral features, our study highlighted that anger expression, insomnia, and auditory hallucinations were significant independent predictors of violent episodes. These dynamic variables reflect acute symptomatic exacerbations and disturbed behavioral states closely linked to imminent violent behavior. This aligns with previous research emphasizing that acute psychotic and affective symptoms are highly predictive of immediate risk (9, 39). Conversely, psychomotor retardation and increased illness insight were identified as protective, suggesting that reduced psychomotor activity and greater self-awareness may inhibit impulsive aggressive responses, consistent with findings by prospective ward-based findings (40) and outpatient prediction studies (41).

Our integrated hierarchical model demonstrated substantial improvements in predictive performance, achieving an AUC of 0.8741, significantly surpassing both the static (AUC = 0.7953) and dynamic (AUC = 0.8003) models alone. The optimal fusion parameter (*α* = 0.37) indicated a stronger predictive contribution from dynamic features, suggesting the temporal sensitivity of these variables is crucial for precise short-term prediction. This hierarchical integration approach echoes similar strategies adopted in other fields of psychiatric risk prediction, where combining stable historical data with dynamic clinical observations significantly enhances predictive accuracy (13, 42, 43).

The present study also underscores the potential clinical utility of machine learning approaches in psychiatric risk assessment. Compared to traditional clinical judgment alone, our findings highlight the benefits of systematically combining a broad array of static and dynamic patient data. Machine learning can identify subtle patterns and complex interactions that might elude conventional assessment methods, thus providing clinicians with objective, data-driven tools to enhance decision-making processes (44–46). Notably, unstructured clinical judgment in violence risk assessment is prone to evaluator bias and shows limited inter-rater agreement (40). Meta-analytic evidence indicates that structured or algorithmic methods outperform clinician judgment when predicting violent outcomes. Consequently, automated machine-learning tools can offer consistent, data-driven risk estimates that reduce subjective variability and complement clinicians’ decisions.

Despite these promising results, several limitations should be acknowledged. Firstly, our findings should be interpreted as demonstrating strong associations rather than definitive causal links. This limitation stems mainly from our reliance on predictive machine-learning algorithms, which focus on correlations rather than causal mechanisms, and is compounded by the retrospective nature of the dataset (47, 48). Future studies employing prospective or real-time data collection methods could offer stronger validation of predictive models (49). Secondly, the generalizability of our findings might be limited, as our patient cohort was sourced from a single psychiatric institution in Liaoning Province. Thus, multicenter studies encompassing broader geographical and cultural diversity are needed to further validate our hierarchical predictive framework. Thirdly, although our dynamic data collection was systematic, the weekly assessment frequency may miss finer-grained temporal dynamics. Real-time or more frequent monitoring using digital health technologies could improve the model’s temporal precision and sensitivity (44). A further limitation is that both the overall sample and the number of violent cases were small. Small training sets make machine-learning models prone to overfitting and inflated effect estimates, and a pronounced class imbalance (few violent cases) can bias predictions toward the non-violent majority (40). Larger, more balanced multi-center cohorts with external validation are therefore needed to improve robustness and generalizability.

Future research directions should consider integrating biological markers such as neuroimaging or genetic data, which might further improve predictive accuracy and elucidate underlying neurobiological mechanisms of violent behavior in schizophrenia patients (13, 50). Moreover, implementing real-time monitoring systems in clinical practice, possibly leveraging wearable devices or digital behavioral tracking technologies, may provide clinicians with timely alerts and facilitate preemptive interventions (51).

Overall, this study demonstrates that a hierarchical machine learning model integrating static clinical information with dynamic behavioral observations has a clear advantage in predicting short-term violence risk among hospitalized patients with schizophrenia. The model shows that dynamic indicators contribute more and identifies that symptoms such as anger expression, insomnia, and auditory hallucinations are closely related to the occurrence of violent incidents. These results provide a basis for adopting data-driven, dynamic risk assessments in future clinical practice.

## Data Availability

The raw data supporting the conclusions of this article will be made available by the authors, without undue reservation.
